# Mapping the oral microbiome in children with oral diseases using next-generation sequencing: a systematic review

**DOI:** 10.1080/20002297.2026.2723644

**Published:** 2026-09-10

**Authors:** Marah Alsaid, Manikandan Ekambaram, Nicholas C. K. Heng, Noren Nor Hasmun

**Affiliations:** a Sir John Walsh Research Institute, Faculty of Dentistry, University of Otago, Dunedin, New Zealand; b Faculty of Dentistry, Applied Science Private University, Amman, Jordan

**Keywords:** Oral microbiome, next-generation sequencing, 16S rRNA gene, oral disease, children, systematic review

## Abstract

**Background:**

The oral microbiome is integral to paediatric oral health and disease. Next-generation DNA sequencing (NGS) technology enables culture-independent profiling of microbial communities, yet findings remain inconsistent due to methodological and population heterogeneity.

**Objective:**

This review synthesised NGS-based evidence comparing the oral microbiome of children/adolescents with oral diseases and healthy controls.

**Design:**

The protocol was registered in PROSPERO (CRD42025618407). PubMed, Medline, Embase, Scopus and Web of Science were searched for peer-reviewed studies (2015–2025). Eligible studies used 16S rRNA gene sequencing to compare children/adolescents (0–18 years) with oral diseases and healthy controls. Two reviewers independently screened studies, extracted data and assessed risk of bias using validated tools. Findings were synthesised narratively due to heterogeneity.

**Results:**

From 4,767 records, 61 studies were included. Most were cross-sectional designs conducted across diverse populations and conditions. Differences in community composition were more consistent than changes in alpha diversity, which varied by disease stage and sampling site. Shifts toward anaerobic, metabolically active communities were observed. Dental caries was consistently linked to acidogenic genera/species, whereas fluorosis, halitosis and gingivitis showed functional shifts involving proteolytic, sulphur-metabolising and anaerobic bacteria.

**Conclusion:**

Paediatric oral diseases are more strongly associated with condition- and niche-specific compositional alterations than with uniform diversity changes.

## KEY MESSAGES

Studies consistently identified microbial biomarkers associated with oral disease and health:

• Caries-associated taxa: *Streptococcus mutans*, *Scardovia wiggsiae*, *Lactobacillus salivarius*, *Prevotella histicola*, *Selenomonas* spp., *Veillonella* spp., *Porphyromonas* spp., the genus *Mogibacterium* and *Actinomyces graevenitzii*.
• Health-associated taxa: *Neisseria subflava*, *Haemophilus parainfluenzae*, *Rothia dentocariosa*, *Fusobacterium periodonticum*, *Capnocytophaga* spp., the genus *Lautropia* and *Corynebacterium matruchotii*. While other associations included *Kingella denitrificans* and *Gemella sanguinis* in fluorosis, *Leptotrichia wadei* and *Peptostreptococcus stomatis* in halitosis and *Streptococcus gordonii* and *Streptococcus australis* in healthy oral surfaces post-intervention.
• Functional analysis (e.g. KEGG pathways and WGS) revealed enrichment of genes involved in carbohydrate metabolism and acid production in caries groups.

## Introduction

The human oral cavity harbours one of the most diverse microbial communities in the body, comprising over 700 bacterial species that play vital roles in oral and systemic health [1,2]. These microbial communities play essential roles in maintaining oral homeostasis and influence systemic health through immune modulation, metabolic activity and barrier function [3,4]. In children, the oral microbiome undergoes particularly dynamic changes. Tooth eruption, dentition transitions, dietary shifts and maturation of the immune system create rapidly changing ecological niches that shape microbial colonisation and succession [5]. During early life, oral microbial communities are less stable and more sensitive to environmental pressures, meaning that changes may follow different trajectories from those seen in adults [6]. In health, this ecosystem exists in a dynamic but stable balance that supports normal oral function and contributes to systemic wellbeing through immune modulation and metabolic activity. Disturbance of this balance, known as ‘dysbiosis’, is now recognised as central to shaping susceptibility to common diseases such as dental caries, gingivitis and enamel defects [7–9]. The concept of oral disease has therefore shifted from a pathogen-centered model to an ecological framework in which dysbiosis reflects changes in community composition, interactions and function driven by environmental pressures such as diet, pH, inflammation and host immunity [9]. Evidence suggests that the early-life oral microbiome may not only reflect but also drive the onset and progression of these diseases [4,10].

Traditional studies of the oral microbiome have relied heavily on culture-based techniques and targeted molecular assays, which are limited in their ability to capture the full complexity and diversity of oral microbial communities [3]. The advent of next-generation DNA sequencing (NGS) technologies, particularly high-throughput sequencing of the ‘gold standard’ 16S rRNA gene, has revolutionised oral microbiome research by enabling comprehensive, culture-independent profiling of bacterial communities which facilitated the identification of both commensal and pathogenic taxa and revealed previously unrecognised shifts in microbial composition associated with oral health and disease in children [3,11].

Despite these advances, significant gaps remain in our understanding of the paediatric oral microbiome. Published studies vary widely in sampling methods, sequencing platforms, bioinformatics pipelines and analytic approaches, resulting in inconsistent findings and challenging direct comparison across studies [12]. Furthermore, most oral microbiome research has focused on adults, while children, whose oral environments and disease risks are distinct, remain understudied [13]. To the best of our knowledge, there is a lack of comprehensive synthesis focused specifically on NGS-based studies of the paediatric oral microbiome across a broad range of oral conditions. Consequently, clinicians and researchers lack a clear, paediatric-specific overview of how oral microbial communities are structured in health, how they adapt and reorganise in disease, how methodological choices influence reported outcomes, and where the major gaps in scientific evidence remain.

Therefore, this systematic review aimed to synthesise the existing literature on NGS-based studies of the paediatric oral microbiome in the context of oral disease. Specifically, we sought to: (1) describe the characteristics and methodological approaches of included studies, (2) synthesise microbial and functional patterns across diverse disease contexts, (3) evaluate consistency and risk of bias in the evidence base and (4) identify priorities for future research. By systematically mapping the available evidence, this review aimed to inform both clinical practice and future research directions in paediatric oral health.

## Materials and methods

### Protocol and registration

This systematic review was conducted in accordance with the Preferred Reporting Items for Systematic Reviews and Meta-Analyses (PRISMA) guidelines [14]. The protocol was prospectively registered with PROSPERO (Registration ID: CRD42025618407).

### Eligibility criteria

Eligibility criteria were prespecified in the registered protocol using the PICO framework. Original, peer-reviewed studies published in English from 2015 onward were eligible if they involved human participants aged 0–18 years and investigated the oral microbiome using next-generation sequencing (NGS) approaches, specifically 16S rRNA gene sequencing. Included studies were required to examine one or more oral disease conditions and include a healthy paediatric control group, with clearly described sampling, sequencing and analytical methods. Studies were excluded if they involved adults exclusively, animal or *in vitro* models, used methods other than 16S rRNA NGS approach, lacked appropriate paediatric controls, or provided insufficient methodological detail. Reviews, conference abstracts, editorials, or unpublished manuscripts were also excluded.

### Information sources and search strategy

Systematic searches were performed in PubMed, MEDLINE, EMBASE, Scopus and Web of Science databases. Reference lists of included articles and relevant reviews were screened to identify additional studies. The search strategy was developed and refined in collaboration with a subject librarian at the University of Otago. The final database search was conducted on 22 January 2026. Search terms combined both Medical Subject Headings (MeSH) and keywords tailored for each database. A combination of ‘oral microbiome’, ‘oral microbiota’, ‘oral bacteria’, ‘next-generation sequencing’, ‘16S rRNA’ and ‘metagenomics’. The full reproducible search strategy is provided in the supplementary material.

### Study selection

**Figure 1.**
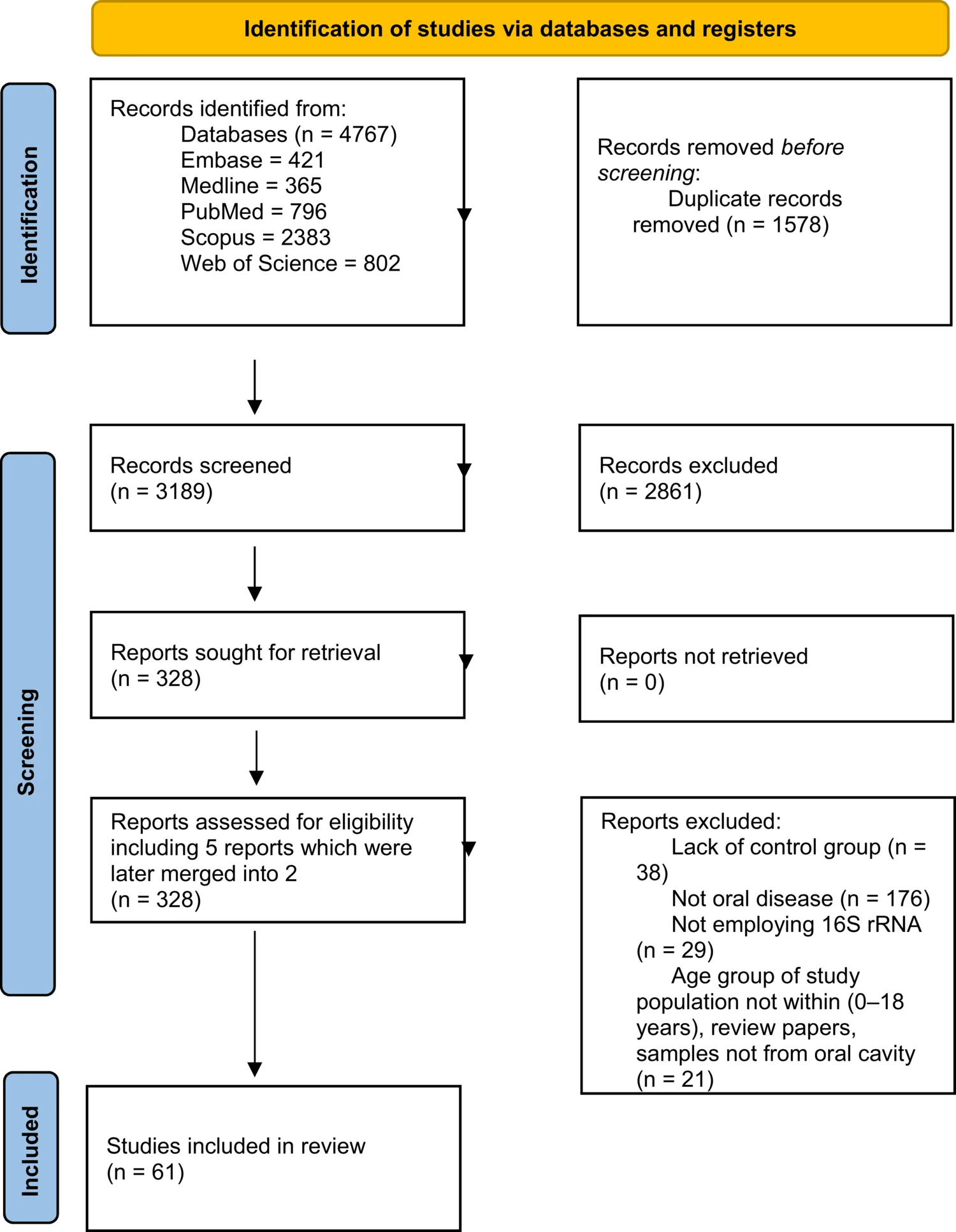
PRISMA flow diagram for study selection. Flow diagram shows the selection of studies that were retrieved, screened and selected for inclusion.

Two reviewers (MAS and NNH) independently performed title and abstract screening using the Rayyan online application [15]. Eligible studies underwent full-text screening independently against the predefined criteria. No machine-learning prioritisation or automated exclusion features were used. Disagreements were resolved by discussion and consensus. The study selection process and reasons for exclusion are presented in a PRISMA flow diagram (Figure 1).

### Data extraction

A standardised data extraction form was used independently by two reviewers (MAS and NNH). Extracted information included: study identifiers (authors, year of publication, country, study design, sample size); participant characteristics (age range, health or disease status and comparison groups); sample type and collection methods; NGS methodologies (targeted regions and sequencing platforms); bioinformatics pipelines (primary sequence-processing workflow and taxonomic databases used); microbial composition outcomes (dominant phyla and genera and differentially abundant taxa); diversity metrics (alpha diversity indices and beta diversity measures);. Where multiple publications reported findings from the same cohort, data were merged to avoid duplication. Missing or unclear information was recorded as ‘not reported’ and not inferred. Study findings were extracted and synthesised descriptively as reported by the original authors, including direction and statistical significance of associations where available, without quantitative pooling.

### Risk of bias assessment

Methodological quality and risk of bias were assessed independently by two reviewers (MAS and NNH). Randomised controlled trials were evaluated using the Revised Cochrane risk-of-bias tool (RoB 2) [16]. Observational and non-randomised studies were assessed using the Joanna Briggs Institute (JBI) critical appraisal checklists appropriate to each study design (cross-sectional and cohort). Non-randomised intervention studies were assessed using the JBI checklist for quasi-experimental designs [17]. Disagreements were resolved through discussion and consensus. Overall risk of bias was categorised as low risk, some concerns, or high risk and summarised visually using the *robvis* tool [18]. Risk of bias assessments informed interpretation of findings but were not used as exclusion criteria.

Reported outcomes primarily included microbial composition, alpha diversity indices (e.g. Shannon diversity, Chao1 richness, Simpson index, Faith's phylogenetic diversity), beta diversity metrics (e.g. Bray–Curtis dissimilarity, weighted and unweighted UniFrac distances) and differentially abundant taxa between comparison groups.

### Data synthesis

Given substantial clinical and methodological heterogeneity, a narrative synthesis approach was employed. Studies were grouped by oral disease type, and key characteristics and outcomes were tabulated and summarised descriptively, focusing on microbial composition, alpha diversity, beta diversity and disease-associated taxa. Taxonomic findings were primarily compared at the phylum and genus levels to maximise comparability. Quantitative meta-analysis was not performed because outcome measures and analytical approaches were not sufficiently comparable across studies.

### Reporting bias and certainty of evidence

Reporting bias and certainty of evidence were assessed qualitatively by examining the completeness and consistency of outcome reporting across studies. Quantitative assessments of reporting bias and formal certainty grading (e.g. funnel plots or the Grading of Recommendations Assessment, Development and Evaluation [GRADE] approach) were not performed because the evidence base was dominated by heterogeneous observational microbiome studies and synthesised narratively, precluding meaningful quantitative evaluation.

## Results

### Search and selection process

A total of 4,767 records were identified through database searching (Embase: 421; Medline: 365; PubMed: 796; Scopus: 2,383; Web of Science: 802). After the removal of 1,578 duplicates, 3,189 records remained for screening. Title and abstract screening excluded 2,861 records, leaving 328 reports for full-text assessment (including one from manual search). No reports were unretrievable. Full-text review resulted in the exclusion of 264 reports for the following reasons: lack of control group (*n* = 38), not oral disease (*n* = 176), not employing 16S rRNA methods (*n* = 29), or other reasons (age group not in range, review papers, protocols, or non-oral samples; *n* = 21). Sixty-four reports were eligible for inclusion. After merging three reports from De Jesus et al [19–21]. as one and two from Wang et al [22,23]. as one (where multiple reports described the same cohort), a total of 61 studies were included in the qualitative synthesis (Figure 1).

### Characteristics of included studies

The characteristics of the 61 included studies are summarised below and detailed in Table 1. The majority of included studies were cross sectional in design (*n* = 46) [24–69], followed by longitudinal cohort studies (*n* = 11) [7,9,70–78]. Interventional designs were less common, comprising one randomised controlled trial [79] and two non-randomised intervention studies [80,81], while one study used a mixed observational design [82].

**Table 1.** Summary of paediatric oral microbiome studies investigating associations between oral conditions and bacterial community composition using 16S rRNA gene sequencing. The table reports study design and population characteristics, oral condition and sample type, sequencing region and platform, bioinformatics pipelines (primary processing workflow; taxonomy database; functional prediction tools, where applicable), diversity metrics with reported group-level differences and taxa identified as increased or decreased in disease/condition groups relative to controls as described by the original authors.

Authors	Study design, country	Sample size (groups); age range	Oral condition	Sample type	16S region & sequencing platform	Bioinformatics pipeline (primary pipeline; taxonomy database; functional tool (if any)	Diversity metrics (direction if reported)	Increased taxa in disease	Decreased taxa in disease
Alia-García, E. et al [41]	Cross-sectional, Spain	42; 4–12 years	Dental caries (ECC)	Unstimulated saliva	V3–V4; Illumina MiSeq	Galaxy-based custom workflow; SILVA 119;	Alpha: Shannon, Chao1; Beta: PCoA	Not specified	Not specified
Blostein, F. et al [73]	Longitudinal cohort, USA	189 (99 ECC, 90 controls); 2 months–5 years	Dental caries (ECC)	Unstimulated Saliva	V4; Illumina MiSeq	DADA2; HOMD v15.2	Alpha: Shannon, Chao1; Beta: community clustering approaches reported	*Streptococcus mutans; Scardovia wiggsiae*	*Neisseria; Haemophilus parainfluenzae; Fusobacterium periodonticum*
Blufstein, A. et al [57]	Cross-sectional, Austria	46 (24 gingivitis, 22 controls); 7–12 years	Gingivitis	Unstimulated saliva	V3–V4; Illumina MiSeq	DADA2, SILVA v138.1	Alpha: Chao1, Shannon (higher in gingivitis); Beta: Bray–Curtis (PCoA, PERMANOVA reported significant differences)	*Selenomonas, Fusobacterium nucleatum; ASVs from Campylobacter, Streptococcus, Dialister, Leptotrichia*	*Haemophilus, Rothia*
Chen, L. et al [29]	Cross-sectional, China	20; 3–5 years	EBS	Supragingival plaque	V3–V4; Illumina MiSeq	QIIME 1.9.1 (UPARSE); RDP Classifier; SILVA	Alpha: Chao1, ACE, Simpson, Shannon; Beta: Unweighted UniFrac PCoA	*Actinomyces naeslundii*	Not specified
Chen, T. et al [37]	Cross-sectional, China	13 (8 caries, 5 controls); 6–18 years	Dental caries	Stimulated saliva + oropharyngeal swabs	V3; Illumina MiSeq	UPARSE v7.0.1001 (97% OTU), RDP Classifier, QIIME 1.8.0	Alpha: Chao1, Shannon; Beta: Unweighted UniFrac, UPGMA, PCA, PCoA (group separation)	*Proteobacteria and Neisseria*	*Firmicutes (especially Streptococcus)*
Chen, W. et al [69]	Cross-sectional, China	80 (40 caries, 40 controls); 6–8 years	Dental caries	Unstimulated saliva and supragingival plaque	V1–V3; pyrosequencing; Roche 454 GS FLX+	Mothur v1.31.2, OTU clustering (97%), SILVA 106, Proteome Discoverer 1.3, Mascot, GO analysis	Alpha: Chao, ACE, Shannon, Simpson indices (alpha); Good's coverage; Beta: Bray–Curtis PCoA, ANOSIM, NMDS (group separation)	*Dialister, Selenomonas, Actinomyces, Mogibacterium, Streptococcus mutans*	*Capnocytophaga, Fusobacterium, Haemophilus, Porphyromonas, Desulfuromonadales*
Chen, Y. et al [28]	Cross-sectional, China	50 (17 SECC, 15 EBS, 18 controls); 2–6 years	Dental caries, EBS	Supragingival plaque	V3–V4; Illumina HiSeq	UPARSE (97%), QIIME, SILVA v132, Mothur, PICRUSt	Alpha: Shannon, Simpson, ACE, Chao1 (EBS group higher richness; BS lowest diversity); Beta: Unweighted UniFrac, NMDS, ANOSIM (group separation)	*Porphyromonas, Selenomonas_3, Fusobacterium* and *Leptotrichia in caries; Selenomonas_3, Capnocytophaga, Actinomyces and uncultured Lentimicrobiaceae linked to EBS*	*Lautropia Abiotrophia*
Cherkasov, S. et al [53]	Cross-sectional, Russia	18 (8 asthma without caries, 10 asthma with caries); 3–6 years	Bronchial asthma with and without dental caries	Supragingival plaque	V3–V4; Illumina MiSeq	PEAR, USEARCH, UCHIME, RDP, NCBI, QIIME2, MicrobiomeAnalyst	Alpha: Good's coverage, Chao1, ACE; Beta: Bray–Curtis dissimilarity, PCoA	*Veillonella (caries); Prevotella loescheii, P. histicola, Kingella oralis, Haemophilus haemolyticus (caries + asthma)*	*Neisseria oralis*
de Jesus, V. et al. [65]	Cross-sectional, Canada	176 (88 S-ECC, 88 controls); 2–6 years	Dental caries (S-ECC)	Supragingival plaque, unstimulated saliva (2021 only)	V4; Illumina MiSeq	QIIME2; Greengenes; PICRUSt2	Shannon/Faith's PD/OTUs mixed (reduced in plaque only); Bray–Curtis/UniFrac; beta separation (site-dependent, S-ECC distinct)	*Prevotella, Porphyromonas, Veillonella, S. mutans, C. albicans*	*Rothia, Haemophilus.*
Dinis, M. et al [52]	Cross-sectional, USA	47; 4–14 years	Dental caries	Unstimulated saliva	V4; Illumina MiSeq	QIIME 1.9.1 (UCLUST); HOMD	Alpha: Shannon index; Beta: Weighted UniFrac, (PCoA)	*High S. mutans groups: increased acidogenic species (V. dispar, Megasphaera, S. intermedius, Prevotella, Actinomyces)*	*Low S. mutans: V. rogosae, Alloprevotella, Haemophilus*
Dzidic, M. et al [9]	Longitudinal cohort, Sweden	90; 3 months–9 years	Dental caries	Unstimulated and stimulated saliva	V3–V4; Illumina MiSeq	USEARCH, CD-HIT (97% similarity, HOMID, BLAST	Alpha: Shannon, Chao1; Beta: CCA, Adonis	*Streptococcus cristatus (significantly higher at 3, 24 mo in caries group), Streptococcus mutans higher at 7 y in caries group*	*S. dentisani* higher in caries-free (by qPCR, not significant)
Eriksen, C. et al [72]	Longitudinal cohort, Sweden	260; 2–5 years	Dental caries	Unstimulated saliva	V3–V4; Illumina MiSeq	DADA2 (R packages); SILVA	Shannon/Richness mixed; Bray–Curtis; beta separation	*Prevotellaceae, Megasphaera, Centipeda*	*Peptococcus*
Eriksson, L. et al [77]	Longitudinal cohort, Sweden	63 (37 caries, 26 controls); 17–19 years	Dental caries	Stimulated saliva, supragingival plaque	V3–V4; Illumina MiSeq; PacBio RS II SMRT (V1–V8, for subset)	Illumina: ProbeSeq (HOMINGS); QIIME v1.8.0 with UCLUST (97% OTU), HOMD BLAST; PacBio: Custom pipeline, BLAST vs HOMD at 98.5% similarity	Alpha: Richness; Beta: PCoA, PCA, LEfSe, PLS (group separation by caries status in saliva; not in plaque)	*Scardovia wiggsiae, Streptococcus mutans, Bifidobacterium longum, Leptotrichia sp. HOT498, Selenomonas spp (saliva)*	*Mycoplasma orale, Porphyromonas sp. HOT278, Fusobacterium nucleatum subsp. Polymorphum (saliva)*
Funahashi, K. et al [35]	Cross-sectional, Japan	10 (6 CLP, 4 controls); 7–15 years	CLP (with/without dental caries)	Supragingival plaque	V1–V3; Illumina MiSeq	QIIME, MG-RAST, SparCC; HOMD; VFDB, MvirDB	Alpha: Shannon, OTU count; Beta: PCoA, ANOSIM	*Streptococcus salivarius, Streptococcus gordonii, Prevotella pallens Actinomyces johnsonii*	Not specified
Grier, A. et al [76]	Longitudinal cohort, USA	56 children (36 developed ECC, 20 remained caries-free); 1–5 years	Dental caries (ECC)	Unstimulated saliva (177 saliva samples)	V1–V3; Illumina MiSeq	QIIME1.9.1, QIIME2 v2018.11 (DADA2), Greengenes	Alpha: Shannon, Faith's; Beta: Weighted UniFrac, PCoA, PERMANOVA (ecological instability precedes ECC)	*Rothia mucilaginosa, Streptococcus spp., Kingella sp., Peptostreptococcus sp., Corynebacterium sp., Selenomonas sp.*	*Veillonella parvulamarkers*
Han, R. et al [58]	Cross-sectional, China	136 (93 ECC, 43 controls); 3–6 years	Dental caries (ECC), iron deficiency anemia (IDA), EBS	Unstimulated saliva	V3–V4; Illumina MiSeq	QIIME v1.9.1, UCLUST, Greengenes, PICRUSt	Alpha: Chao1, Shannon, Simpson, PD whole tree, observed species (ECC > CF; IDA-ECC > NIDA-ECC; EBSCF < NEBS-CF); Beta: Unweighted/weighted UniFrac, PCoA, PERMANOVA	*ECC (overall): Neisseria, Streptococcus, Prevotella* *IDA-ECC: Bacillus, Moraxella, Rhodococcus* *NIDA-ECC: Neisseria* *EBSCF: Neisseria*	*Actinomyces, Rothia, Corynebacterium*
Hurley, E. et al [30]	Cross-sectional, Ireland	138 (68 S-ECC, 70 controls) < 5 years	Dental caries (S-ECC)	Unstimulated saliva (all), deep dentinal lesion samples (S-ECC group)	V4–V5; Illumina MiSeq	FLASH v1.2.8, QIIME v1.9.1, USEARCH v8.0.1623 (OTU clustering at 97%), mothur v1.36.1, SPINGO v1.3	Alpha: Chao1, Observed Species, PD whole tree, Shannon, Simpson (reduced in caries lesions); Beta: PCA, PCoA, Bray–Curtis, weighted/unweighted UniFrac (group separation)	*Scardovia wiggsiae, Prevotella histicola, Prevotella denticola (caries active); Streptococcus mutans, Prevotella, Scardovia, Bifidobacterium (S-ECC dentine)*	*Capnocytophaga, Leptotrichia*
Jiang, W. et al [40].	Cross-sectional, China	58; 8–22 years	CLP	Unstimulated saliva	V3–V4; Illumina NovaSeq	QIIME2(DADA2, Vsearch, FLASH); Greengenes; PICRUSt2	Alpha: Chao1, ACE, observed species, Simpson, Shannon, Pielou Beta: Weighted UniFrac, PCoA, ADONIS (group separation observed)	*Neisseria, Haemophilus, Porphyromonas, Granulicatella*	*Rothia, Veillonella, Pauljensenia*
Jung, M. et al [26]	Cross-sectional, Germany	64 (29 MIH, 35 controls); 7–17 years	MIH	Supragingival plaque	V4; Illumina MiSeq	DADA2 (R packages); SILVA	Shannon/Pielou/Observed mixed (reduced with MIH severity); Morisita–Horn; no separation	*Streptococcus spp. (ASV0055), Mannheimia sp.*	*Bergeyella sp.*
Karched, M. et al [27]	Cross-sectional, Kuwait	40 (20 caries-active, 20 controls); 7–9 years	Dental caries	supragingival plaque	V3–V4; Illumina MiSeq	ProbeSeq (HOMINGS); BLAST	Shannon/Chao1; PCA/ANOSIM/PCoA; no separation	*Leptotrichia, Veillonella, Kingella, S. sanguinis, L. shahii, S. mutans, TM7[G-1] sp. HOT_346*	*Neisseria, L. hongkongensis, Abiotrophia defectiva, Veillonella dispar, Haemophilus parainfluenzae*
Kasimoglu, Y. et al [54]	Cross sectional, Turkey	198 (99 twin pairs: 49 MZ, 50 DZ); 3–15 years	Dental caries	Stimulated saliva	V3–V4; Illumina MiSeq	QIIME2, Deblur, vsearch, USEARCH, CASAVA, PEAR, SILVA 132_99,	Alpha: ASVs, observed species; Beta: Bray–Curtis, weighted/unweighted UniFrac, PCoA (MZ twins more similar than DZ twins)	Not specified	Not specified
Kim, B.-S. et al [70]	Longitudinal cohort, South Korea	24 (12 dentine caries, 12 controls); 6–13 years	Dental caries	Stimulated saliva	V1–V3; Roche 454 GS Junior	USEARCH (97% OTU), EzTaxon-e database, Mothur, PICRUSt	Alpha: Shannon, OTU count (Lower diversity in caries group at follow-up); Beta: PCoA; carbohydrate metabolism higher in caries group	*Streptococcus, Granulicatella, Staphylococcus, Bulleidia*	*Actinomyces, Leptotrichia, Atopobium, Veillonella, Neisseria, Lautropia*
Lee, E. et al [44]	Cross-sectional, Korea	120 (30 caries, 30 controls); 3-6 years (30 caries, 30 controls), 6–12 years	Dental caries	UnstimulatedSaliva, supragingival plaque	V3–V4; Illumina HiSeq	QIIME2, Deblur, SILVA, scikit-learn, machine learning	Alpha: Shannon, Faith's PD, Observed features, Pielou's evenness; Beta: Jaccard distance, PCoA, LEfSe (Significant taxonomic and functional differences by age and caries status)	*Scardovia wiggsiae, Leptotrichia wadei* (caries < 6 y)*; Streptococcus mutans (*caries 6–12y)	*Neisseria oralis, Corynebacterium durum*
Li, K. et al [67]	Cross-sectional, China	60 (30 SECC, 30 controls); 3–6 years	Dental caries (S-ECC)	Unstimulated saliva	V3–V4; Illumina NovaSeq	QIIME 1.9.1 (UPARSE 97% OTU); SILVA138; MetaboAnalyst/KEGG	Alpha: ACE, Chao1, Shannon (higher in SECC); Beta: Weighted/unweighted UniFrac, PCoA, NMDS (group separation)	*Veillonella; Lactobacillus; Lautropia; Aggregatibacter*	*Neisseria; Porphyromonas; Streptobacillus; Absconditabacteriales*
Li, Y. et al [63]	Cross-sectional, China	25 (10 EBS, 15 controls); 4–5 years	EBS	Saliva, supragingival plaque	V3–V4; Illumina (unspecified)	MOTHUR; OTU clustering at 97%	Alpha: Shannon, Simpson, ACE, Chao1 (reduced with EBS); Beta: PCA (EBS plaque more variable)	*Actinomyces, Cardiobacterium, Haemophilus, Corynebacterium, Tannerella, Treponema*	*Campylobacter,* *Streptococcus*
Li, Y. et al [50]	Cross-sectional, China	221 (85 EBS/caries, 26 controls); Not specified (Primary dentition)	Dental caries, EBS	Supragingival plaque, saliva (111 saliva, 110 plaque samples)	V1–V3; 454 Genome Sequencer FLX (Roche)	QIIME, USEARCH61, BLAST, Greengenes	Alpha: Observed species (OTUs), Shannon; Beta: Bray–Curtis, Unweighted UniFrac, binary Jaccard, PCoA, NMDS (group separation); plaque more discriminatory than saliva	*Caries: Gemellales, Granulicatella; Streptococcus, Mogibacterium; Selenomonas, Gemella, Streptobacillus; Haemophilus, Veillonella, Prevotella;* *Pigment: Leptotrichia, Fusobacterium, Rothia, Prevotella, Streptococcus;* *Caries + pigment: Streptococcus, Mogibacterium*	*Leptotrichia*
Lin, X. et al [31]	Cross-sectional, China	90; 3–5 years	Dental caries (S-ECC)	Unstimulated saliva	V3–V4; Illumina NovaSeq	QIIME2 (DADA2, FastTree, MAFFT); HOMD16S	Alpha: Chao1, Shannon, Simpson, Observed species, Faith's PD; Beta: Bray–Curtis, unweighted UniFrac, PCoA (group separation by CA/dmft)	*Scardovia, Selenomonas, Campylobacter*	*Abiotrophia, Lautropia*
Liu, L. et al [74]	Longitudinal cohort, China	28; 8–16 years	CLP (surgical inflammation as outcome)	Unstimulated saliva (pre/post data 56 samples)	V3–V4; Illumina MiSeq	QIIME v1.8.0, OTU clustering (99%), Greengenes 13_8	Alpha: Shannon index, OTU richness; Beta: Unweighted UniFrac, PCoA, PCA, Random Forest (group separation)	*Tannerella, Porphyromonas, Gemella, Prevotella* *Porphyromonas sp., Porphyromonas nigrescens, Porphyromonas intermedia*	*Veillonella parvula, Neisseria sp. 86.7%*
Liu, M. et al [34]	Cross-sectional, China	78 (55 CP, 23 non-CP); 6–14 years	Dental caries (with/without CP)	Supragingival plaque	V3–V4; Illumina MiSeq	USEARCH (OTU clustering/Mothur; Greengenes	Alpha: Shannon index, OTU count (not significant) Beta: NMDS, PERMANOVA, correlation networks (group separation)	*Prevotella; Fusobacterium; Porphyromonas; Saccharibacteria; Porphyromonas intermedia, Actinomyces tannerae, Fusobacterium nucleatum*	*Leptotrichia; Capnocytophaga*
Luo, S. et al [38]	Cross-sectional, China	46; 9–18 years	Dental fluorosis	supragingival plaque	V1–V9; PacBio Sequel	QIIME2/DADA2; HOMD; PICRUSt2	Shannon/Chao1/Faith's PD reduced; UniFrac; beta separation	*Prevotella sp_HMT_304, Gemella sanguinis*	*Streptococcus oralis subsp. dentisani, Kingella denitrificans, Capnocytophaga gingivalis*
Manning, S. et al [47]	Cross-sectional, Thailand	177; 3 years	Dental caries (ECC)	Stimulated saliva	Probe-based (Forsyth Institute, Harvard University)	QIIME 1.9.1 (OTU picking); Taxonomy: Not reported;	Alpha: Shannon; Beta: Bray–Curtis (PCoA), (group separation)	*Oribacterium sinus, Alloprevotella* and other caries-associated taxa	*Prevotella, Veillonella, Neisseria, Rothia, Leptotrichia*
Manzoor, M. et al [36]	Cross-sectional, Finland	617 (208 caries, 409 controls); 9–12 years	Dental caries	Unstimulated saliva	V3–V4; Illumina HiSeq	Mothur v1.35.1, UCHIME, Silva v119	Alpha: Shannon index, Inverse Simpson index; Beta: Bray–Curtis dissimilarity, PCoA, PERMANOVA	*Paludibacter and Labrenzia*	*Phenylobacterium, Macrococcus*
Nagai, N. et al [46]	Cross-sectional, Japan	16; 1–12 years	Dental caries	Supragingival plaque (32 samples)	V3–V4; Illumina MiSeq	QIIME v1.9.1, USEARCH v6.1.544, SILVA 119, OTU clustering at 99%	Alpha: Observed OTUs, Shannon index; Beta: Weighted UniFrac distance, PCoA	*Actinomyces, Cardiobacterium, Kingella, Veillonella.*	*Lautropia and Neisseriaceae*
Nomura, Y. et al [51]	Cross-sectional, Myanmar	13; 9–13 years	Dental caries	Supragingival plaque	V3–V4; Illumina MiSeq	R Bioconductor Microbiome package, EzBioCloud 16S	Alpha: Shannon, Simpson, Chao1, ACE; Beta: Bray–Curtis, PCA (group separation)	*Streptococcus mutans, Veillonella dispar, Lactobacillus mucosae, Neisseria bacilliformis, Parascardovia denticolens, Prevotella multisaccharivorax; Firmicutes, Bacteroidetes*	*Rothia mucilaginosa; Proteobacteria*
Ortiz, S. et al [59]	Cross-sectional, USA	85 children (64 caries, 21 controls); 2–12 years	Dental caries	Unstimulated saliva	V3–V4; Illumina (HOMINGS at Forsyth Institute)	HOMINGS, ProbeSeq	Alpha: richness, abundance described, (no alpha metric value in main text); Beta: Not directly named (group comparison, fold-change, p/FDR for beta, not ordination)	*Caries active: Leptotrichia, Lactococcus, Treponema; Boys: Neisseria flavescens, Rothia aeria, Haemophilus pittmaniae; Girls: Lactococcus lactis, Alloprevotella HOT 473, Veillonella parvula, Selenomonas sp.,*	Not specified
Paul, B. et al [43].	Cross-sectional, USA	20; 6–13 years	Dental caries (SDF treatment failure)	Saliva and supragingival plaque	V3–V4; Illumina MiSeq	QIIME2, DADA2, LEfSe, ALDEx2, HOMD, PICRUSt	Alpha: Observed taxa, Shannon, Simpson (reduced in non-responders); Beta: Bray–Curtis, PCA/PCoA, UniFrac, PERMANOVA (group separation)	Non-responders: *Granulicatella, Leptotrichia, Tannerella, Lachnospiraceae; Streptococcus mutans* and *Rothia* more abundant in SDF and non-responders	*Saccharibacteria*
Qudeimat, M. et al [33]	Cross-sectional, Kuwait	128 (64, caries, 64 controls); 6–9 years	Dental caries (S-ECC)	Supragingival plaque	V3–V4; HOMINGS via Illumina MiSeq	ProbeSeq (BLAST), Primer-7	Alpha: Shannon, Chao1; Beta: Euclidean distance-based PCoA	*Leptotrichia shahii, Prevotella melaninogenica, Veillonella dispar, Streptococcus mutans*	*Corynebacterium matruchotii, Lautropia mirabilisClear*
Raksakmanut, R. et al [78]	Longitudinal cohort, Thailand	30 (10 white spot lesions, 10 cavitated caries; 10 controls); 1–2 years	Dental caries	Unstimulated saliva	V3–V4; Illumina MiSeq	QIIME2 (DADA2); eHOMD	Alpha: Shannon, Chao1, Pielou evenness; Beta: Unweighted UniFrac, PCoA, ANOSIM, PERMANOVA (group separation)	Not specified	*Prevotella nanceiensis; Leptotrichia sp. HMT 215; Prevotella melaninogenica; Campylobacter concisus*
Ren, W. et al [68]	Cross-sectional, China	20 (10 halitosis, 10 controls); 4–5 years	Halitosis	Tongue coating, stimulated saliva (final analysis 19 samples)	V1–V3; 454 GS FLX Titanium	Mothur, QIIME, HOMD, KEGG/eggNOG	Alpha: Observed OTUs, CatchAll, Chao1 (increased in halitosis tongue not saliva); Beta: Weighted UniFrac; PCA on OTUs, co-occurrence networks	*Leptotrichia wadei, Peptostreptococcus stomatis (tongue); Prevotella shahii (saliva)*	Not specified
Ribeiro, A. et al [48]	Cross-sectional, Brazil	13 (46 samples: 24 AWSL, 22 sound); 12 years	Dental caries ( AWSL)	Supragingival plaque	V1–V2; Illumina MiSeq	BLASTN-based pipeline, QIIME, HOMD v13.2, HOMDextended 1.1, Greengenes Gold	Alpha: Chao1, Observed species, Shannon index (reduced in AWSL and with high-carbohydrate diet); Beta: Unweighted/weighted UniFrac, PCoA (group separation)	*Streptococcus sp. Oral_Taxon_065, Corynebacterium matruchotii, Actinomyces viscosus, Actinomyces sp. Oral_Taxon_175/178/877, Prevotella nigrescens, Dialister micraerophilus, Eubacterium_XI G1 infirmum*	*Streptococcus gordonii, Streptococcus australis, Streptococcus sp. Oral_Taxon_058/071/F11, Enterobacter sp. (str. 638/cloacae), Yersinia mollaretii, Centipeda sp. Oral_Taxon_D18*
Richards, V. et al [75]	Longitudinal cohort, USA	55; 2–7 years	Dental caries	Supragingival plaque	V3–V4; Illumina MiSeq	QIIME (v1.9.0), HOMINGS, ProbeSeq	Alpha: Shannon; Beta: Bray–Curtis dissimilarity, db-RDA (group separation)	*Streptococcus mutans, Scardovia wiggsiae, Parascardovia denticolens, Lactobacillus salivarius* (dentin caries)	*Streptococcus sanguinis, Neisseria, Lautropia*
Roca, C. et al [56]	Cross-sectional, USA	88 (15 caries, 31 gingivitis; some overlap; 37 controls);7–18 years	Dental caries, gingivitis	Saliva (stimulated, unstimulated)	V1–V2; Illumina MiSeq	QIIME2 (DADA2); HOMD	Alpha: Faith's PD ↑ in disease/stimulated; Beta: Bray–Curtis (PERMANOVA)	*Capnocytophaga, Aggregatibacter (caries); Peptostreptococcus (gingivitis)*	Not specified
Simon-Soro, Á. et al [7]	Longitudinal cohort, UK (Scotland) & Spain	33 (14 caries, 5 developed caries, 14 controls); 4–5 years	Dental caries	Unstimulated saliva	V1–V3; Roche GS-FLX Titanium	UCLUST (97% OTU), QIIME, RDP classifier	Alpha: OTU richness, Shannon Index; Beta: PCA, Bray–Curtis, PCoA, FastUnifrac	*Aggregatibacter, Lactococcus, Selenomonas, Lactobacillus; Streptococcus higher at baseline in those who later developed caries*	*Rothia, Catonella, Corynebacterium, Capnocytophaga, Granulicatella, Cardiobacterium*
Skelly, E. et al [80]	Interventional (non-randomised), Australia	103 (69 intervention, 34 untreated); 7–14 years	Dental caries	Stimulated saliva	V4; Illumina MiSeq	QIIME2 v2018.8, EMP protocol, Deblur, Greengenes v13.8, HOMD v15.1, SILVA 132	Alpha: Shannon, Chao1, observed species (reduced with preventive intervention); Beta: Bray–Curtis, Jaccard, weighted/unweighted UniFrac, PCoA, ANOSIM, PERMANOVA	*Lactobacillus salivarius, L. reuteri, L. gasseri, Prevotella multisaccharivorax, Parascardovia denticolens, Mitsuokella HMT 131, Streptococcus mutans; Corynebacterium and Leptotrichia* (untreated only)	Not specified
Starr, J. et al [55]	Cross-sectional, USA	254 (154 HIV- infected, 100 HIV-exposed- uninfected); 10–22 years	Denta caries, periodontitis	Subgingival plaque	V3–V4; Illumina MiSeq	DADA2 (v1.4), QIIME, HOMD (v14.51), Naive Bayesian classifier	Alpha: Shannon, Simpson; Beta: Not reported, differences in taxa assessed using regression models	Periodontitis-associated taxa showed expected associations only in HIV- exposed- uninfected, not HIV- infected	*Corynebacterium durum, Corynebacterium matruchotii, Abiotrophia defectiva* (in HIV- infected vs HIV- exposed- uninfected)
Sulyanto, R. et al [81]	Interventional (non-randomised), USA	42 (16 plaque pre/post-SDF, 13 SDF dentin, 13 control dentin); Mean age 4.3 years	Dental caries (ECC)	Supragingival plaque; dentin	V1–V3; Illumina MiSeq	Mothur, BLAST	Alpha: Not directly named, but abundance and species richness; Beta: NMDS, Bray–Curtis, PERMANOVA (Subsurface: group separation)	SDF increased *S. salivarius, S. gordonii, A. odontolyticus, L. delbrueckii, S. sobrinus, R. aeria, S. mitis* group	*S. mutans, F. nucleatum, Porphyromonas CW034*
Tu, Y. et al [32].	Cross-sectional, China	55 (15 ECC, 22 SECC, 18 controls); Mean age 45.1 ± 9.6 months	Dental caries (S-ECC, ECC)	Unstimulated saliva	V3–V4; Illumina MiSeq/NovaSeq	QIIME, FLASH, UPARSE (99% OTU), SILVA 138/UNITE 8.0,	Alpha: Shannon index, Chao index;Beta: Bray–Curtis PCoA, ANOSIM, PERMANOVA, PLS-DA (cluster structure differed)	*TM7x, Selenomonas, Fusobacterium and Prevotella*	*Streptococcus*
Wang, Q. et al [61].	Cross-sectional, China	42 (14 mild, 19 moderate/severe, 9 controls);12–14 years	Dental fluorosis	Unstimulated saliva	V4; Illumina HiSeq	QIIME2 (DEBLUR ), FLASH, Greengenes 13_8, BLASTN (NCBI)	Alpha: Shannon, Observed OTUs, Faith's phylogenetic diversity; Beta: Bray–Curtis, weighted/unweighted UniFrac, Jaccard, PCoA, ANOSIM, (significantly altered in moderate/severe dental fluorosis)	*M&S: Firmicutes, Proteobacteria; Streptococcus, Streptococcus mitis, Gemella parahaemolysans, Lactococcus lactis, Fusobacterium nucleatum*	*Bacteroidetes; Prevotella, Porphyromonas; Prevotella melaninogenica, Schaalia odontolytica, Bifidobacterium dentium*
Wang, Y. et al. [22,23]	Mixed observational, China	44 (21 S-ECC, 23 controls); 3–5 years	Dental caries (S-ECC)	Unstimulated saliva	V1–V9; PacBio RS II	Mothur/QIIME; SILVA; HUMAnN2	Shannon/Simpson/Chao/ACE mixed; PCA/NMDS/PCoA; beta separation (caries more variable, controls more stable)	*Lactobacillus, Mogibacterium, Dialister, Prevotella, Veillonella, S. mutans, Selenomonas*	*Neisseria, Haemophilus, Rothia, S. rubneri*
Wang, Y. et al [42]	Cross-sectional, China	30, 2–4 years	Dental caries (S-ECC)	Supragingival plaque	V3–V4; Illumina MiSeq	QIIME2 (DADA2); SILVA	Alpha: Observed species, Shannon; Beta: PCoA (unweighted UniFrac)	*Prevotella, Lactobacillus*	*Rothia, Neisseria*
Wirth, R. et al [45]	Cross-sectional, Hungary	28; 15–18 years	Gingivitis	Supragingival plaque and saliva	V3–V4; Illumina MiSeq, Illumina NextSeq	QIIME2, Kraken2, MEGAN6	Alpha: Shannon index; Beta: Bray–Curtis dissimilarity, PCA, UPGMA clustering (group separation)	*Fusobacterium; Campylobacter; Akkermansia; Treponema*	*Lautropia; Kingella; Neisseria; Actinomyces; Rothia; Rothia dentocariosa; Haemophilus parainfluenzae*
Wu, Y. et al [66]	Cross-sectional, China	88 (44 rampant caries, 44 controls); 2–5 years	Dental caries (Rampant)	Supragingival plaque	V3–V4; Illumina NovaSeq	QIIME 1.9.1 (UPARSE 97% OTU); SILVA v138	Alpha: Chao1, Shannon Beta: Unweighted UniFrac, PCoA, NMDS, ANOSIM (group separation observed)	*Proteobacteria, Campylobacterota, Neisseria, Fusobacterium, Streptococcus mutans, Porphyromonas gingivalis, Bifidobacterium acidifaciens*	*Veillonella, Firmicutes, Leptotrichia*
Yang, X. et al [39]	Cross-sectional, China	30 (15 caries, 15 controls); 7–9 years	Dental caries	supragingival plaque unstimulated saliva (90 samples: 3 types per child)	V1–V9; PacBio Sequel	QIIME v1.8.0, USEARCH v5.2.236, UCLUST (97% OTU), HOMD	Alpha: Simpson, Chao1, ACE, Shannon indices (saliva lower diversity than plaque); Beta: Weighted UniFrac, NMDS, Adonis (key taxa differ by group)	*Deciduous: Streptococcus mutans, Veillonella dispar, Parascardovia acidifaciens*	*Deciduous: Streptococcus noxia, Corynebacterium matruchotii* *Permanent: Streptococcus noxia, Leptotrichia, Selenomonas*
Yang, Z. et al [49]	Cross-sectional, China	36 (18 high caries, 18 controls); 5 years	Dental caries	Unstimulated saliva	V3–V4; Illumina NovaSeq	QIIME (USEARCH/UPARSE 97% OUT); SILVA 128; PICRUSt2, BugBase	Alpha: Shannon, Simpson, Chao, ACE, PD_whole_tree Beta: Bray–Curtis, weighted UniFrac, unweighted UniFrac, PCoA, ANOSIM (no separation)	*Selenomonas, Bifidobacterium, Dialister, Olsenella, Anaeroglobus, Propionibacterium (genus), uncultured Prevotella_7, Propionibacterium species*	*Prevotella*
You, Y. et al [24]	Cross-sectional, China	44 (25 with GT, 19 controls); 2–10 years	Geographic tongue (GT)	Lingual swab (lesion, normal mucosa and healthy controls)	V3–V4; Illumina MiSeq	UPARSE/QIIME2; RDP; PICRUSt2	Shannon/Simpson/Chao1/ACE/Observed mixed (reduced in GT lesions); UniFrac; beta separation	*Prevotella oris, Catonella, Oribacterium, Bacillus, Prevotella nanceiensis*	*Streptobacillus*
Zhang, D. et al [62].	Cross-sectional, Japan	138; 61, 6-7 years and 77, 11–12 years	Dental caries	Tongue coating	V1–V2; Ion PGM system	UPARSE (97% OTU), BLAST against eHOMD v15.1	Alpha: Richness (observed OTUs), no significant difference by caries; Beta: Unweighted UniFrac (beta diversity), PCoA, PERMANOVA; differences by caries status and age	*Veillonella parvula, Streptococcus parasanguinis, Prevotella histicola, Actinomyces graevenitzii*	*Neisseria subflava, Porphyromonas pasteri, Fusobacterium periodonticum, Haemophilus parainfluenzae, Streptococcus oralis subsp. dentisani*
Zhang, Q. et al [79]	Randomised Controlled Trial, China	41 (20 probiotic, 21 controls); 3–6 years	Early childhood caries (ECC)	Unstimulated Saliva and dental plaque	V3–V4; Illumina MiSeq	QIIME2 (DADA2); SILVA	Alpha: Shannon, Chao1, Faith’s PD; Beta: Bray–Curtis, UniFrac (weighted & unweighted), PCoA (stability higher with probiotic)	*Streptococcus mutans, Candida albicans*	*Firmicutes, Streptococcus* (non-mutans), *Gemella, Granulicatella, ‘healthy genera’;* *Proteobacteria, Neisseria, Bifidobacterium, Catonella* were reduced with probiotic
Zhang, Y. Fang, J. et al [60]	Cross-sectional, China	217 (95 SECC;132 controls); 2–6 years	Dental caries (S-ECC)	Supragingival plaque	V3–V4; Illumina HiSeq	QIIME, Mothur, SILVA, PICRUSt	Alpha: Chao1, ACE, Shannon, Simpson indices (higher in SECC); Beta: NMDS, UniFrac (unweighted), PCoA (group separation)	*SECC S. mutans-low: Leptotrichia, Selenomonas, Prevotella_7 as biomarkers; SECC S. mutans-high: Porphyromonas, Selenomonas_3*	*Lautropia, Streptococcus, Neisseria, Bergeyella, Abiotrophia*
Zhang, Y., Yu, R. et al [64]	Cross-sectional, China	69 (21 EBS, 48 controls); 3–4 years	EBS	Plaque and Unstimulated saliva	V3–V4; Illumina MiSeq	QIIME 1.9.1 (UPARSE); HOMD v15.2; PICRUSt	Alpha: Chao (higher in EBS plaque), Shannon (lower in EBS plaque); saliva: no difference; Beta: Bray–Curtis, weighted UniFrac, PCoA, ANOSIM (group separation in plaque)	*Pseudomonas fluorescens; Actinomyces sp._HMT_169; Leptotrichia sp._HMT_212; Aggregatibacter sp._HMT_898 (BTS plaque)*	*Streptococcus; Rothia; Actinomyces naeslundii; Corynebacterium matruchotii; iron metabolism genes enriched in EBS microbiome*
Zhang, Y., Zhu, C. et al [71]	Longitudinal cohort, China	20 (10 halitosis, 10 controls); 3–4 years	Halitosis	Tongue coating	V3–V4; Illumina MiSeq	FLASH, Trimmomatic, UPARSE v7.1, HOMD, QIIME, PICRUSt	Alpha: Shannon, Chao1 ↑ at follow-up in both groups; Beta: Weighted UniFrac, PCoA, ANOSIM	*P. melaninogenica, Saccharibacteria TM7_G-1, Actinomyces sp._HMT_180*	Not specified
Zheng, L. et al [25]	Cross-sectional, China	38 (74 samples); 3–6 years	Dental caries, EBS	Supragingival plaque	V3–V4; Illumina MiSeq	UPARSE; SILVA	Alpha: Sobs, ACE, Simpson, Shannon, Coverage( ↓ in disease); Beta: PCA, db-RDA, network analysis (group-separated)	*Porphyromonas, Fusobacterium (EBS−/caries+); Veillonella (caries + groups); Actinomyces, Cardiobacterium (EBS+/caries+)*	*Lautropia, Pesudopropionibacteriumin (EBS+/caries−);*

Abbreviations: ECC, early childhood caries; SECC, severe early childhood caries; EBS, enamel black stain; MIH, molar–incisor hypomineralisation; GT, geographic tongue; CLP, cleft lip and/or palate; CP, Cleft palate; SDF, silver diamine fluoride; AWSL, active white spot lesion; MZ, monozygotic; DZ, dizygotic; OTU, operational taxonomic unit; ASV, amplicon sequence variant; QIIME, Quantitative Insights Into Microbial Ecology; PCoA, principal coordinates analysis; PERMANOVA, permutational multivariate analysis of variance.

Most studies were undertaken in Asia (*n* = 43), with China being the largest single contributor (Table 1). Additional studies were conducted across Europe, North America, Australia, Brazil, Canada, Kuwait, Myanmar and Turkey (Table 1).

The median sample size was 50 children, with an inter-quartile range (IQR) of 73. Whereas most studies enrolled between 30 and 103 participants, a few large-scale studies included over 200 children [36,55,60,72].

The included studies covered children and adolescents across a wide paediatric age range (approximately 3 months to 18 years). Studies focused on children in the preschool and early school-age periods up to 6;years (*n* = 28) [7,25,28–32,42,47,49,50,53,58,60,64–68,71–73,75,76,78,79,81,82], while school-aged children and adolescents between 6 and 18 years were investigated in 24 studies [26,27,29,33–40,43,45,48,51,55–57,61,62,69,70,74,77,80]. Eight studies examined broader or mixed paediatric age ranges, extending across multiple developmental periods (e.g. from 3 months up to 15 years) [9,24,41,44,46,52,54,59]. Studies also reported specific mean ages with standard deviations [32,81].

### Quality assessment

**Figure 2.**
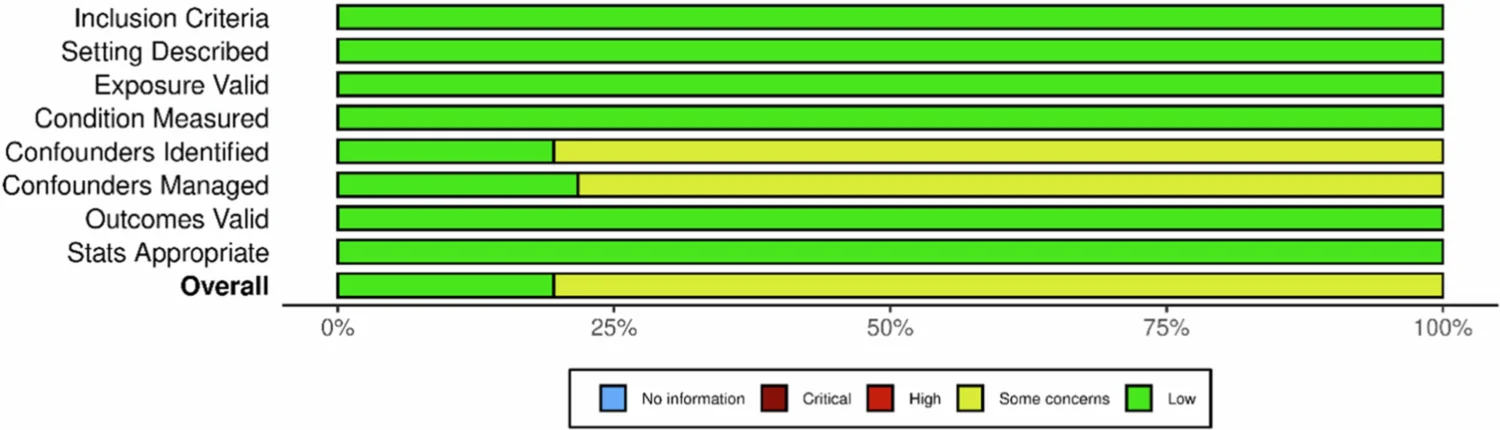
Summary of risk of bias across cross-sectional studies. The stacked bar chart shows the proportion of cross-sectional studies rated as low risk, some concerns; none was considered a high risk across each methodological domain. Outcome measurement and statistical analysis were most consistently rated as low risk, whereas identification and control of confounding showed the greatest variability.

Overall risk of bias was generally low to moderate, with the most frequent concerns relating to confounding, particularly in cross-sectional designs. Longitudinal and interventional studies more commonly accounted for confounding but showed occasional concerns related to follow-up and baseline comparability. Traffic-light plots showing domain-level judgments for individual studies are presented by design in Supplementary Figures S1–S4. A summary plot for cross-sectional studies, the largest study group, is shown in Figure 2.

### Oral health conditions studied

Dental caries dominated the literature and was investigated in various forms comparing caries-active groups with caries-free controls, such as early childhood caries (ECC) [41,47,73,76,79,81], severe ECC (S-ECC) [30–33,42,60,65,67,82] and active caries lesions [7,9,27,36,37,39,43,44,46,48,49,51,52,54,59,62,66,69,70,72,75,77,78,80]. Additional disease categories included gingival and periodontal conditions, primarily gingivitis [45,56,57], tooth pigmentation disorders, most commonly extrinsic black tooth stains, assessed either independently [29,63,64], or in combination with dental caries [25,28,50]. Developmental enamel defects were also represented, including molar-incisor hypomineralisation (MIH) [26] and dental fluorosis [38,61]. Smaller numbers of studies investigated halitosis, diagnosed using volatile sulphur compound measurements [68,71] and oral mucosal conditions specifically geographic tongue [24]. In addition, several studies examined oral microbiome alterations associated with systemic conditions including cerebral palsy [34], human immunodeficiency virus (HIV) infection [55], bronchial asthma [53] and iron deficiency anaemia [58], as well as craniofacial conditions with oral manifestations particularly cleft lip and palate (CLP) [35,40,74].

### Sample types, collection methods, DNA extraction and sequencing approaches

Across the 61 included studies utilising 16S rRNA amplicon sequencing, saliva-based samples were the most frequently collected. Unstimulated saliva, primarily obtained by spitting into sterile tubes, drooling, or sterile swabbing techniques, was reported in 19 studies [7,31,32,36,40,41,49,52,57–59,61,67,72–74,76,78,82]. Stimulated saliva, collected by chewing paraffin wax, was used in 5 studies [37,47,54,70,80], while a combination of both unstimulated and stimulated saliva was collected in 2 studies [9,56]. Plaque samples were also common with supragingival plaque collected in 19 studies [25–29,33–35,38,42,46,48,51,53,60,63,66,75,81] and subgingival plaque in 1 study [55]. Methods included the use of sterile curettes, excavators, paper points, toothpicks, cotton swabs, or toothbrush scraping. Additionally, 10 studies collected both saliva and plaque samples concurrently [39,43–45,50,64,65,69,77,79]. One study collected unstimulated saliva (by swabbing) together with deep dentinal lesion samples (by a sterile spoon excavator) [30]. Less frequently, studies employed tongue coating samples collected by scraping (*n* = 2) [62,71], or collected tongue coating along with saliva (*n* = 2) [24,68].

A wide range of commercial DNA extraction kits were employed through studies. Qiagen-based kits predominated (*n* = 17), encompassing stool, blood/tissue, soil, microbiome and low-biomass/micro-scale applications [24,26,27,29,33,43,48–50,56,63,65,73,74,78,81,82], Other commonly used commercial kits included Tiangen (*n* = 8) [28,32,37,58,60,61,66,68], Epicentre/MasterPure (*n* = 6) [7,47,52,55,57,75], Omega Bio-tek/E.Z.N.A. (*n* = 6) [25,31,38,39,64,71] among others.

For sequencing, the Illumina MiSeq platform was most commonly used in 39 studies [9,24–27,29,30,32–35,37,41–43,45,46,48,51–58,63–65,71–81], with additional studies employing the Illumina HiSeq (*n* = 5) [28,36,44,60,61] or NovaSeq (*n* = 5) [31,40,49,66,67] systems. Some studies used alternative platforms, such as the now-obsolete 454 GS-FLX or GS Junior (*n* = 5) pyrosequencing systems [7,50,68–70], PacBio RS-II or Sequel platforms (*n* = 3) [38,39,82] and the Ion Torrent PGM [62] system. Two studies utilised the HOMINGS platform (The Forsyth Institute) for probe-based identification of oral taxa [47,59].

The V3–V4 region of the 16S rRNA gene was by far the most frequently-targeted hypervariable region (*n* = 37) [9,24,25,27–29,31–34,36,40–46,49,51,53–55,57–60,63,64,66,67,71,72,74,75,77–79], followed by studies focusing on the V1–V3 region (*n* = 8) [7,35,50,68–70,76,81] and the V4 region (*n* = 6) [26,52,61,65,73,80]. Other targeted regions included V1–V2 (*n* = 3) [48,56,62], V1–V9 (full-length) (*n* = 3) [38,39,82] and V4–V5 [30].

### Bioinformatic processing, diversity analyses and statistical methods

Across the included studies, bioinformatic processing of 16S rRNA sequencing data was heterogeneous. Early studies predominantly employed operational taxonomic unit (OTUs)-based pipelines, most commonly clustering sequences at 97% sequence identity using UPARSE [24,25,28,29,37,62,64,66,67,71] or UCLUST [7,39,50,52,53,58,70,77], with 99% OTU identity reported in a small number of studies [32,46,61,74]. Several OTU-based studies employed Mothur [83] either as a primary pipeline or for downstream diversity analyses and OTU processing, including chimera removal and alpha-diversity estimation [28,30,34,36,49,60,63,68–70,81,82].

More recent studies increasingly adopted amplicon sequence variant (ASV)–based denoising approaches, primarily using DADA2 [84] within QIIME2 [85] or directly in R [26,31,38,40,42,43,45,51,55–57,65,72,73,76,78,79], while Deblur [86] was applied in a smaller subset of QIIME2-based workflows [44,54,61,80].

Taxonomic assignment was performed using curated reference databases, most commonly SILVA [25,26,28,29,32,36,41,42,44,46,49,54,57,60,66,67,69,72,79,80,82], the Human Oral Microbiome Database (HOMD) [87] or its extended versions [9,31,35,38,39,43,48,52,53,55,56,62,64,68,71,73,78,81], Greengenes [34,40,50,58,61,65,74,76], or EzBioCloud [51]. Species- or genus-level identification using probe-based or BLAST-based approaches, including HOMINGS or ProbeSeq, was reported in selected studies either as a primary or complementary analytical strategy [27,33,47,59,75,77].

Alpha diversity (within a single microbial community or sample, reflecting both species richness and evenness) was reported in all studies using established indices, most frequently Shannon and Chao1, with additional metrics including Simpson, ACE, observed OTUs/ASVs, Faith's phylogenetic diversity, Pielou's evenness, Good's coverage, or richness estimates depending on the pipeline used (as detailed per study in Table 1). Beta diversity (differences in microbial community composition between samples or groups) was similarly widely assessed, most commonly using Bray–Curtis dissimilarity or unweighted and weighted UniFrac distances, visualised through principal coordinate analysis (PCoA), non-metric multidimensional scaling (NMDS), or principal component analysis (PCA), and statistically evaluated using permutational multivariate analysis of variance (PERMANOVA), analysis of similarities (ANOSIM), permutational multivariate analysis of variance using distance matrices (ADONIS), or related multivariate approaches (Table 1). Statistical testing across studies predominantly relied on non-parametric comparisons, including Wilcoxon rank-sum or Mann–Whitney *U* tests and Kruskal–Wallis tests. Statistical significance was generally defined at *p* < 0.05, with *p*-values often adjusted for multiple comparisons using false discovery rate (FDR) correction.

Beyond taxonomic and diversity-based analyses, several studies additionally incorporated functional prediction or complementary omics analyses, including PICRUSt or PICRUSt2-based inference [24,28,38,40,49,65,70,71], metagenomic or metatranscriptomic analyses [35,68,82], or integrated machine learning frameworks for classification or [41,44,48,49,54,55,58,59,64,65,73,78].

### Microbial diversity and composition

Across studies, the paediatric oral microbiome was dominated by several core phyla: Firmicutes, Bacteroidetes, Proteobacteria, Actinobacteria and Fusobacteria. Frequently reported genera in both health and disease included *Streptococcus, Veillonella, Neisseria, Rothia, Prevotella, Haemophilus, Actinomyces, Granulicatella, Leptotrichia, Porphyromonas* and *Fusobacterium* (Table 1).

Caries-associated communities were reported to show higher abundance of acidogenic and aciduric taxa, including *Streptococcus mutans, Scardovia wiggsiae, Lactobacillus* spp. and members of the genera *Veillonella, Prevotella, Selenomonas, Dialister* and *Bifidobacterium* [7,30,31,33,36,42–44,49,51,52,59,65–67,69,70,73–75,77,80,82]. Health-associated or caries-free profiles were reported to have higher abundance of the genera *Rothia*, *Neisseria*, *Haemophilus*, *Corynebacterium*, *Capnocytophaga*, *Lautropia* and *Kingella* [27,33,37,39,48,49,59,62,69,73,75,77,78,82]. Several studies further demonstrated lesion- or site-specific signatures, with dentine and subsurface plaque showing stronger disease discrimination than saliva [30,75,81]. Alpha diversity findings were heterogeneous. Several studies reported lower diversity in caries or severe early childhood caries (S-ECC) [30,43,65,80], whereas others reported higher richness or no significant differences, particularly in early disease [27,41,49], or severe and advanced lesions [58,60,67]. Longitudinal studies showed that early microbiome profiles predicted later caries development [7,9,72,73,76,78], and interventional studies demonstrated microbiome restructuring after probiotic or silver diamine fluoride (SDF) treatment [43,79–81].

Gingivitis was consistently associated with enrichment of anaerobic and inflammation-associated genera such as *Fusobacterium, Campylobacter, Selenomonas, Treponema, Dialister* and *Porphyromonas*[45,56,57], while healthy controls showed higher prevalence of the genera *Rothia, Neisseria, Kingella, Haemophilus* and *Actinomyces*[45,57]. Some studies reported higher alpha diversity in gingivitis [57], whereas others observed overlaps between groups despite clear compositional shifts [45,56].

Dental fluorosis groups showed depletion of several putative health-associated taxa, including members of the genera *Kingella*, *Capnocytophaga*, *Schaalia, Actinomyces*, *Bifidobacterium* and *Streptococcus oralis*–related taxa, alongside enrichment of taxa more often linked to acidogenic or dysbiotic states, such as species belonging to the genera *Streptococcus* (including non-mutans streptococci), *Gemella*, *Prevotella*, *Lactococcus* and *Fusobacterium*[38,61]. Oral bacterial communities in MIH cases exhibited largely conserved overall community structure; however, specific ASVs assigned to the *Streptococcus* genus and *Mannheimia* sp. were associated with MIH severity and caries risk, with reduced diversity observed in more severe cases of MIH [26].

Extrinsic black tooth staining indicated distinct but relatively stable communities enriched in members of the genera *Actinomyces, Cardiobacterium, Corynebacterium, Tannerella* and iron- or sulphur-associated taxa [29,63,64]. Some studies showed that black staining was associated with lower caries prevalence and different community network structures compared with healthy and caries groups [25,28,50,58].

Halitosis was characterised by enrichment of tongue-associated anaerobes including members of the genera such as *Leptotrichia, Peptostreptococcus, Prevotella* and *Actinomyces,* together with members of the phylum Saccharibacteria (TM7) [68,71], with enrichment of sulphur-metabolism and hydrogen sulphide–producing pathways [68,71]. Network analyses showed a shift of microbial hubs from saliva in healthy children to tongue coating in halitosis [68].

Geographic tongue showed lesion-specific enrichment of *Prevotella oris,* and members of the genera *Catonella, Oribacterium* and *Bacillus,* with depletion of the genus *Streptobacillus*[24]. Although alpha diversity was not significantly different, beta diversity showed clear separation between lesions and controls, with predicted functional shifts [24].

Systemic or syndromic conditions modified oral microbiome disease relationships. Children with cerebral palsy and severe caries showed enrichment of members of the genera *Prevotella, Fusobacterium* and *Porphyromonas,* together with members of the phylum Saccharibacteria, with aciduric taxa increasing with lesion severity [34]. HIV-infected youth showed reduced health-associated taxa such as the genus *Corynebacterium* and altered links between the microbiota and periodontitis [55]. Asthma combined with caries was associated with higher abundance of the genus *Veillonella* and the species *Prevotella loescheii* and *Prevotella histicola*, together with reduced *Neisseria oralis* [53]. CLP studies reported differences in salivary or plaque microbiomes with higher abundance of members of the genera *Streptococcus, Prevotella, Porphyromonas, Neisseria* and *Haemophilus*, with depletion of genera such as *Rothia* and *Veillonella* [35,40,74]. Dysbiosis was often more evident at a functional or network level rather than by abundance alone, with altered transcriptional activity, network structure and predicted metabolic pathways in CLP cohorts [35,40]. Pre-operative enrichment of the genera *Porphyromonas*, *Tannerella* and *Prevotella* predicted a higher risk of post-surgical inflammation, whereas *Neisseria* sp. and *Veillonella parvula* were linked to more clinically favourable outcomes [74].

## Discussion

In this systematic review, we synthesised evidence from 61 studies which employed NGS-based 16S rRNA gene sequencing to characterise the oral microbiome across paediatric oral diseases. In accordance with the predefined eligibility criteria, only 16S rRNA gene sequencing studies were included, as it is a well-established approach for bacterial community profiling, supported by comprehensive reference databases and analytical workflows [1,12,83]. Restricting the review to a single sequencing methodology avoided introducing additional complexity that would arise from combining different sequencing technologies. Despite this, the evidence base remained highly heterogeneous, with variation in sampling approaches, targeted hypervariable regions of the 16S rRNA gene, sequencing platforms, bioinformatic pipelines and analytical frameworks, which limited direct comparability across studies. Dental caries, particularly early childhood caries, predominated, whereas other oral and systemic conditions were comparatively under-represented. Across disease categories, alterations in community composition were more consistently observed than changes in alpha diversity, which remained variable. Our findings underscore ecological patterns in paediatric oral dysbiosis while highlighting the need for methodological standardisation and more robust longitudinal evidence.

### Cross-condition synthesis

When considered across the evidence base, several unifying patterns emerged as reported by primary studies. First, the paediatric oral microbiome was dominated by core phyla (Firmicutes, Bacteroidota, Proteobacteria, Actinobacteria and Fusobacteria) and recurrent genera including *Streptococcus*, *Veillonella*, *Neisseria*, *Rothia*, *Prevotella*, *Haemophilus* and *Actinomyces*. Compositional changes were more consistent than diversity changes with significant beta diversity separation and taxonomic restructuring, whereas alpha diversity varied widely. Functional and network-level alterations also often appeared more pronounced than taxonomic differences alone, particularly for pathways related to carbohydrate metabolism, acid stress, sulphur metabolism, host interaction and microbial invasion. Second, many conditions appeared to align along a broader dysbiosis axis. Disease-associated communities were commonly enriched in taxa associated with acidogenic, anaerobic, proteolytic, or inflammation-related ecological states, including mutans streptococci and members of the genera *Scardovia, Lactobacillus, Prevotella, Selenomonas* and *Dialister.* Whereas associations involving the genera *Veillonella*, *Fusobacterium, Campylobacter* and *Porphyromonas* were more variable across oral conditions and studies. In contrast, health-associated communities more often included genera such as *Lautropia, Neisseria* and *Rothia,* while associations involving the genera *Haemophilus, Corynebacterium* and *Kingella* varied according to the oral condition, ecological context, taxonomic resolution and methodological approach. Across conditions such as caries, gingivitis, fluorosis and halitosis, these shifts were frequently accompanied by functional profiles consistent with increased carbohydrate fermentation, proteolysis, sulphur metabolism and anaerobic respiration. MIH, however, generally showed more subtle and less consistent microbial changes, consistent with localised ecological variation rather than a uniform disease-associated dysbiotic pattern. Third, disease discrimination was strongly influenced by ecological niche. Plaque and lesion-specific samples, particularly dentine and subsurface plaque, showed stronger disease separation than saliva, while tongue coating was especially informative for halitosis and mucosal conditions. This highlights the importance of site-specific sampling in oral microbiome research and suggests that biological relevance may be improved when sampling strategies are aligned with the anatomical niche most directly involved in disease processes. Finally, host and environmental factors; including age, diet, oral hygiene, enamel integrity, systemic disease and inflammatory or genetic background substantially modified how dysbiosis manifested, explaining much of the heterogeneity between studies. These themes should be interpreted as qualitative consistencies across heterogeneous studies rather than as pooled estimates.

### Interpretation of microbial patterns across disease contexts

Across multiple studies, caries-associated communities were recurrently enriched for acidogenic and aciduric communities, which are functionally adapted to fermentable carbohydrate environments and low pH, reflecting the classic ecological plaque hypothesis in caries development. In contrast, health-associated profiles were often marked by higher abundances of commensal genera linked to nitrate reduction, oxygen tolerance and ecological stability.

Similarly, gingivitis and early periodontal inflammation in children demonstrated enrichment of anaerobic, inflammation-associated communities that parallel patterns described with adult periodontal dysbiosis [84]. This suggests that early inflammatory dysbiosis in children shares core taxonomic features with adult disease and may represent a continuum rather than a distinct paediatric entity. Developmental enamel defects including MIH and fluorosis displayed more subtle but nevertheless discernible microbiome patterns, suggesting that altered enamel substrates may facilitate site-specific microbial enrichment even when the global bacterial community structure remains stable.

Extrinsic black stain (EBS) harboured distinct microbial signatures enriched for iron- and sulphur-associated microbial communities. Evidence suggests that this phenotype may be associated with lower caries prevalence, raising the possibility that pigmentation reflects a stable ecological configuration rather than pathology [85]. Clinically, this challenges the perception of black stain as purely aesthetic and underscores the importance of distinguishing ecological phenotypes from disease states. Understanding whether such microbial profiles confer resilience against caries could inform preventive strategies and risk assessment in paediatric populations.

Halitosis was consistently associated with tongue-coating anaerobes and sulphur-metabolism pathways, which was consistent with functional drivers of volatile sulphur compound (VSC) production [68,71]. Geographic tongue displayed lesion-specific microbial enrichment, further highlighting niche specificity in non-carious conditions [24]. This reinforces the concept that oral conditions are better defined by specific ecological reorganisation than by global diversity metrics, and that niche sampling (i.e. tongue coating) yields higher disease discrimination than bulk saliva or plaque sampling alone. These findings suggest that diagnostic and research sampling strategies should align closely with the biological niche of interest to improve sensitivity and interpretability.

Systemic or syndromic conditions illustrate how oral microbial ecology is shaped not only by local plaque-host interactions but also by host immune status, inflammatory modulation and systemic physiological constraints. In children with cerebral palsy and severe caries, enrichment of members of the genera *Prevotella*, *Fusobacterium* and *Porphyromonas*, together with members of the phylum Saccharibacteria reflects a combined effect of altered immune function, increased plaque retention due to motor impairment, and chronic low-grade inflammation, creating an environment that favours anaerobic and dysbiotic communities [34]. Asthma, a chronic inflammatory respiratory condition, when present with caries was associated with higher abundance of the genus *Veillonella* and the species *Prevotella loescheii* and *Prevotella histicola*, together with lower abundance of *Neisseria oralis*, consistent with evidence that systemic inflammatory states and airway disease can influence oral microbial composition, possibly via immune-mediated shifts in mucosal surfaces and salivary factors that alter niche suitability for specific taxa [53]. In the case of perinatally acquired HIV infection, depletion of health-associated genera including *Corynebacterium* was observed [55], in line with clinical observations that immunocompromised states are linked to oral dysbiosis and increased susceptibility to opportunistic colonisation and periodontal manifestations. These phenomena reflect a broader paradigm in which the oral microbiome is not an isolated ecosystem but part of an integrated host–microbe network. Such bidirectional interactions between the oral microbiome and systemic health have been documented in the literature [86], highlighting that oral dysbiosis can exacerbate systemic inflammatory pathways and that systemic conditions can alter oral microbial balance through immune modulation and changes in host-microbe interactions [87,88].

### Diversity and functional inference

Alpha diversity findings were heterogeneous across conditions and niches, reinforcing evidence that diversity metrics alone are insufficient markers of paediatric oral disease. This inconsistency likely reflects both biological and methodological variation. Biological factors such as differences in oral niche, disease stage and severity, dentition stage, age, diet and geographic population can influence the composition of the oral microbiome. Similarly, methodological factors such as variation in sampling protocols, DNA extraction methods, primer selection, targeted hypervariable regions, sequencing platforms, sequencing depth, bioinformatic pipelines, diversity metrics and statistical analyses can substantially affect diversity estimates. Therefore, inconsistent findings in alpha diversity should be interpreted cautiously, as they may partly reflect methodological variability rather than true biological dysbiosis. These patterns align with broader systematic evidence in paediatric and adolescent populations showing that community composition, rather than diversity loss alone, underlies caries development and that functional traits such as carbohydrate metabolism drive these associations [5,89].

Heterogeneity was also evident in differential abundance and ‘biomarker’ discovery methods. (e.g. LEfSe, DESeq2, MaAsLin2, non-parametric tests), with inconsistent handling of compositionality, sparsity, multiple-testing correction and covariate adjustment. As a result, reported ‘biomarkers’ should be interpreted cautiously, particularly where confounding factors (diet, oral hygiene, age, caries experience, antibiotic exposure, sequencing batch) were incompletely controlled [90]. This heterogeneity likely contributes to between-study variability in disease-associated taxa and limits direct comparability across conditions and sampling sites. Importantly, most 16S rRNA-based microbiome datasets are compositional (relative abundance constrained to a constant sum), meaning that apparent increases in one taxon can arise from decreases in others even without changes in absolute load; potentially inflating false-positive associations or distorting effect directions [91].

Several included studies incorporated predictive functional inference tools, such as PICRUSt2, to estimate the metabolic potential of microbial communities from 16S rRNA gene sequencing data [24,28,38,40,49,55,65,70,71]. These analyses revealed enrichment of pathways related to carbohydrate metabolism, acid stress response, sulphur metabolism and invasion in diseased states. Machine learning approaches were also applied in a subset of studies to improve disease prediction [41,44,47–49,54,55,58,59,64,65,73,78,81], highlighting the growing emphasis on translating microbiome data into clinically relevant biomarkers [92].

Although predictive functional inference provides valuable insight into the metabolic potential of microbial communities, it remains an indirect approach derived from taxonomic composition. Emerging shotgun metagenomic and metatranscriptomic studies complement 16S rRNA sequencing by enabling species- and strain-level taxonomic resolution together with direct characterisation of functional pathways, transcriptionally active communities, bacteriophage populations and host–microbiome interactions. Recent paediatric studies applying these technologies have identified functional alterations associated with oral diseases that extend beyond taxonomic shifts, including changes in carbohydrate metabolism, arginine biosynthesis, biofilm-related functions, microbial transcriptional activity and bacteriophage community composition [35,93–97]. These approaches should be viewed as complementary to, rather than replacements for 16SrRNA sequencing, extending taxonomic profiling by providing insights into disease pathogenesis.

### Strengths and limitations of this review

This review provides a comprehensive synthesis of paediatric oral microbiome studies using NGS approaches, integrating methodological, ecological and functional perspectives across diverse conditions. All included studies used well-defined healthy or caries-free controls, and several employed within-subject comparisons, strengthening evidence for site-specific microbial shifts.

The substantial methodological heterogeneity across sampling strategies, DNA extraction methods, primer selection, targeted sequencing regions, reference databases and bioinformatic pipelines limited direct comparison between studies and precluded quantitative meta-analysis. In addition, the predominance of cross-sectional study designs restricted causal inference regarding the relationship between microbial alterations and disease onset. Reporting and adjustment for important confounding factors, including diet, antibiotic exposure and oral hygiene, were inconsistent and represented a major source of moderate risk of bias. Furthermore, this review focused exclusively on bacterial communities. Therefore, other components of the oral microbiome, including fungi, viruses, archaea and other eukaryotic microorganisms, were beyond its scope. Increasing evidence suggests that these microbial communities interact with bacteria through physical adhesion, chemical signalling, metabolic exchange and other cross-kingdom relationships that shape oral microbial communities [98]. Consequently, oral microbial ecology is determined not only by bacterial composition, but also by spatial organisation, biofilm architecture, complex interspecies interactions [99,100]. Integrating these components into future microbiome studies is likely to provide a more comprehensive understanding of the ecological and functional mechanisms underlying paediatric oral health and disease. Similarly, advances in shotgun metagenomic and metatranscriptomic sequencing are expanding microbiome research beyond taxonomic characterisation by enabling species-level resolution and functional profiling of microbial communities. Although these approaches were beyond the scope of the present review, they represent an important direction for future paediatric oral microbiome research. Publication bias may have influenced the available evidence, as studies with significant findings are more likely to be published, and restriction to English-language studies and 16S rRNA gene sequencing may have excluded relevant evidence. These limitations highlight the need for more rigorous, standardised and longitudinal paediatric oral microbiome research.

### Implications for clinical practice and research

**Figure 3.**
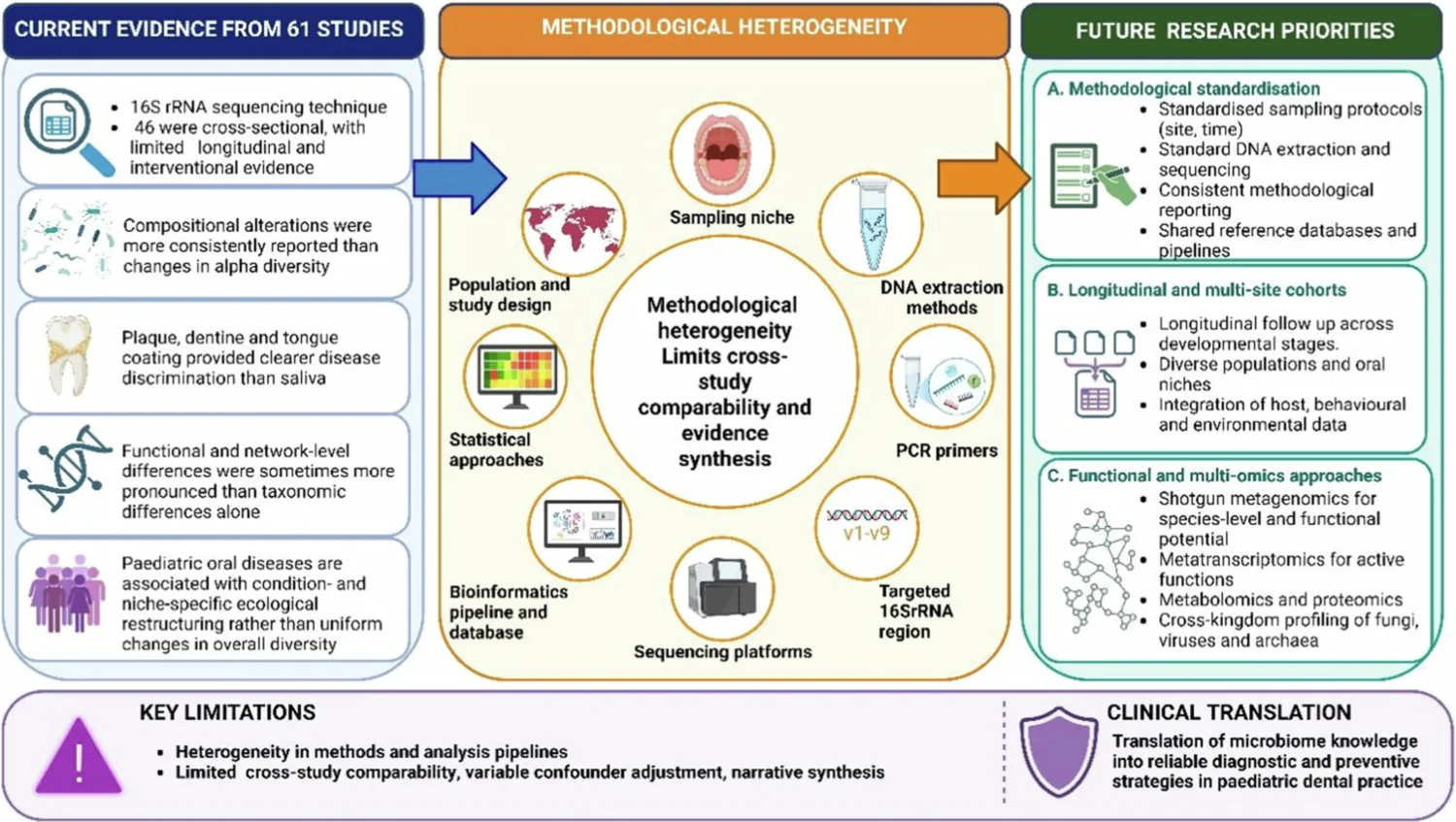
Graphical overview of the current evidence, methodological challenges, limitations and future research priorities in paediatric oral microbiome research. The figure integrates the principal findings of this systematic review with the key methodological challenges and limitations affecting the evidence base, and highlights priorities for future research and clinical translation (created in BioRender).

Future paediatric oral microbiome research should prioritise methodological standardisation to improve reproducibility, facilitate direct comparison between studies and enable quantitative evidence synthesis. Standardised sampling protocols tailored to specific oral niches would enhance diagnostic precision and enable meaningful meta-analyses. Adopting longitudinal designs to capture developmental trajectories from infancy to adolescence. Enhanced reporting of confounding factors and consistent inclusion of healthy control groups will improve the reliability of associations and support the development of targeted preventive and therapeutic strategies. Future paediatric studies should integrate taxonomic profiling with functional multi-omics approaches, ecological network analyses and host-response data to provide a more comprehensive understanding of the ecological and molecular mechanisms underlying paediatric oral diseases. Such advances are likely to improve the identification of disease-associated microbial signatures, facilitate biomarker discovery and support the development of more precise microbiome-based diagnostic and preventive strategies (Figure 3).

## Conclusions

This systematic review demonstrates that paediatric oral diseases are associated with compositional and functional reorganisation of microbial communities rather than uniform changes in overall diversity. Across conditions, disease is characterised by enrichment of anaerobic taxa, loss of health-associated commensals and shifts in functional pathways related to carbohydrate metabolism, acid stress and inflammation. These changes are strongly niche-dependent, with plaque, dentine and tongue coating providing clearer disease signals than saliva. Future well-designed longitudinal studies integrating taxonomic profiling with complementary functional approaches, including shotgun metagenomics and metatranscriptomics, are needed to strengthen the evidence base, improve understanding of disease mechanisms and facilitate translation of microbiome knowledge into reliable diagnostic and preventive strategies in paediatric dental practice.

## Supplementary Material

Supplementary Material RoB_Traffic_light_Supplementary.docx

## Data Availability

Data sharing is not applicable to this article as no new data was created or analysed in this study.
