# Correction

**DOI:** 10.1080/20002297.2026.2743220

**Published:** 2026-10-04

Article title: Mapping the Oral Microbiome in Children with Oral Diseases Using Next-Generation Sequencing: A Systematic Review

Authors: Alsaid, M., Heng, N. C. K., Ekambaram, M., & Hasmun, N. N.

Journal: *Journal of Oral Microbiology*

Bibliometrics**:** Volume 18, Number 01, pages 1—30

DOI**:** http://doi.org/10.1080/20002297.2026.2723644

This article was originally published with an incorrect author sequence:

Marah Alsaid, Manikandan Ekambaram, Nicholas C. K. Heng, and Noren Nor Hasmun

The correct author sequence is:

Marah Alsaid, Nicholas C. K. Heng, Manikandan Ekambaram, and Noren Nor Hasmun

This has now been corrected, and the article has been republished.

