# Supplementary material for: Mapping the oral microbiome in children with oral diseases using next-generation sequencing: a systematic review
Source: J Oral Microbiol. 2026 Sep 10;18(1):2723644. doi: 10.1080/20002297.2026.2723644 (PMC13564157; doi:10.1080/20002297.2026.2723644)
Supplement: Supplementary file 1 [file ZJOM_A_2723644_SM5411.docx]

**Supplementary Figure S1.** Risk-of-bias traffic-light plot for cross-sectional studies.

**Supplementary Figure S2.** Risk-of-bias traffic-light plot for cohort and longitudinal studies.

**Supplementary Figure S3.** Risk-of-bias traffic-light plot for interventional and quasi-experimental studies.

**Supplementary Figure S4.** Risk-of-bias assessment of the randomised controlled trial using RoB 2.
